# Successful treatment of infantile oxysterol 7α-hydroxylase deficiency with oral chenodeoxycholic acid

**DOI:** 10.1186/s12876-021-01749-x

**Published:** 2021-04-13

**Authors:** Yun-Ping Tang, Jing-Yu Gong, Kenneth D. R. Setchell, Wujuan Zhang, Jing Zhao, Jian-She Wang

**Affiliations:** 1 Department of Pediatrics, Jinshan Hospital, Fudan University, Shanghai, 201508 China; 2 Department of Gastroenterology, Qilu Children’s Hospital of Shandong University, Jinan, 250022 Shandong China; 3 Department of Pathology and Laboratory Medicine, Cincinnati Children’s Hospital Medical Center, Cincinnati, OH 45229 USA; 4 The Center for Pediatric Liver Diseases, Children’s Hospital of Fudan University, 399 Wanyuan Road, Shanghai, 201102 China

**Keywords:** Cholestasis, Oxysterol 7α-hydroxylase, *CYP7B1*, Hereditary spastic paraplegia

## Abstract

**Background:**

Deficiency of oxysterol 7α-hydroxylase, encoded by *CYP7B1*, is associated with fatal infantile progressive intrahepatic cholestasis and hereditary spastic paraplegia type 5. Most reported patients with *CYP7B1* mutations presenting with liver disease in infancy have died of liver failure. However, it was recently reported that two patients treated with chenodeoxycholic acid survived. Correlations between the phenotype and genotype of *CYP7B1* deficiency have not been clearly established.

**Case presentation:**

A 5-month-7-day-old Chinese baby from non-consanguineous parents was referred for progressive cholestasis and prolonged prothrombin time from one month of age. Genetic testing revealed compound heterozygous mutations c.187C > T(p.R63X)/c.334C > T(p.R112X) in *CYP7B1*, and fast atom bombardment mass spectrometry analysis of the urinary bile acid confirmed the presence of atypical hepatotoxic 3β-hydroxy-Δ^5^-bile acids. While awaiting liver transplantation she was orally administered chenodeoxycholic acid. Her liver function rapidly improved, urine atypical bile acids normalized, and she thrived well until the last follow-up at 23 months of age. Her 15-year-old brother, with no history of infantile cholestasis but harboring the same mutations in *CYP7B1*, had gait abnormality from 13 years of age. Neurological examination revealed hyper-reflexia and spasticity of the lower limbs. Brain MRI revealed enlarged perivascular space in the bilateral basal ganglia and white matter of frontal parietal.

**Conclusions:**

In summary, these findings highlight that the phenotype of *CYP7B1* deficiency varies widely, even in siblings and that early administration of chenodeoxycholic acid may improve prognosis.

## Background

Congenital bile acid synthesis deficiencies (CBASD) are rare inherited metabolic diseases caused by a number of enzyme defects in the complex pathway of bile acid synthesis. Most are autosomal recessive genetic diseases, accounting for about 1–2% of the children with cholestatic disease [1]. Oxysterol 7α-hydroxylase deficiency [2] caused by *CYP7B1* mutations is also known as congenital bile acid synthesis defect type 3(CBASD3), and is characterized by neonatal cholestasis, and fat-soluble vitamin malabsorption. Most patients with CYP7B1 deficiency presenting with liver disease in infancy succumb to liver failure in early life. However, it was recently reported that two patients treated with chenodeoxycholic acid survived [3, 4]. In addition, hereditary spastic paraplegia type 5 (SPG5), which presents as neurological disease later in childhood or adulthood may also be associated with *CYP7B1* mutations [5–10]. The *CYP7B1* gene that encodes for the enzyme oxysterol 7ɑ-hydroxylase is located on chromosome 8q21.3, and contains 6 exons and 5 introns, 202.6 kb in length. Correlations between the phenotype and genotype of *CYP7B1* deficiency have not been clearly established [11, 12]. Here we describe the clinical features, laboratory examinations and treatment responses of an infant presenting with neonatal cholestasis, and her older brother presenting as SPG5, that further provides insight into this rare congenital bile acid synthesis disorder.

## Case presentation

**Fig. 1:**
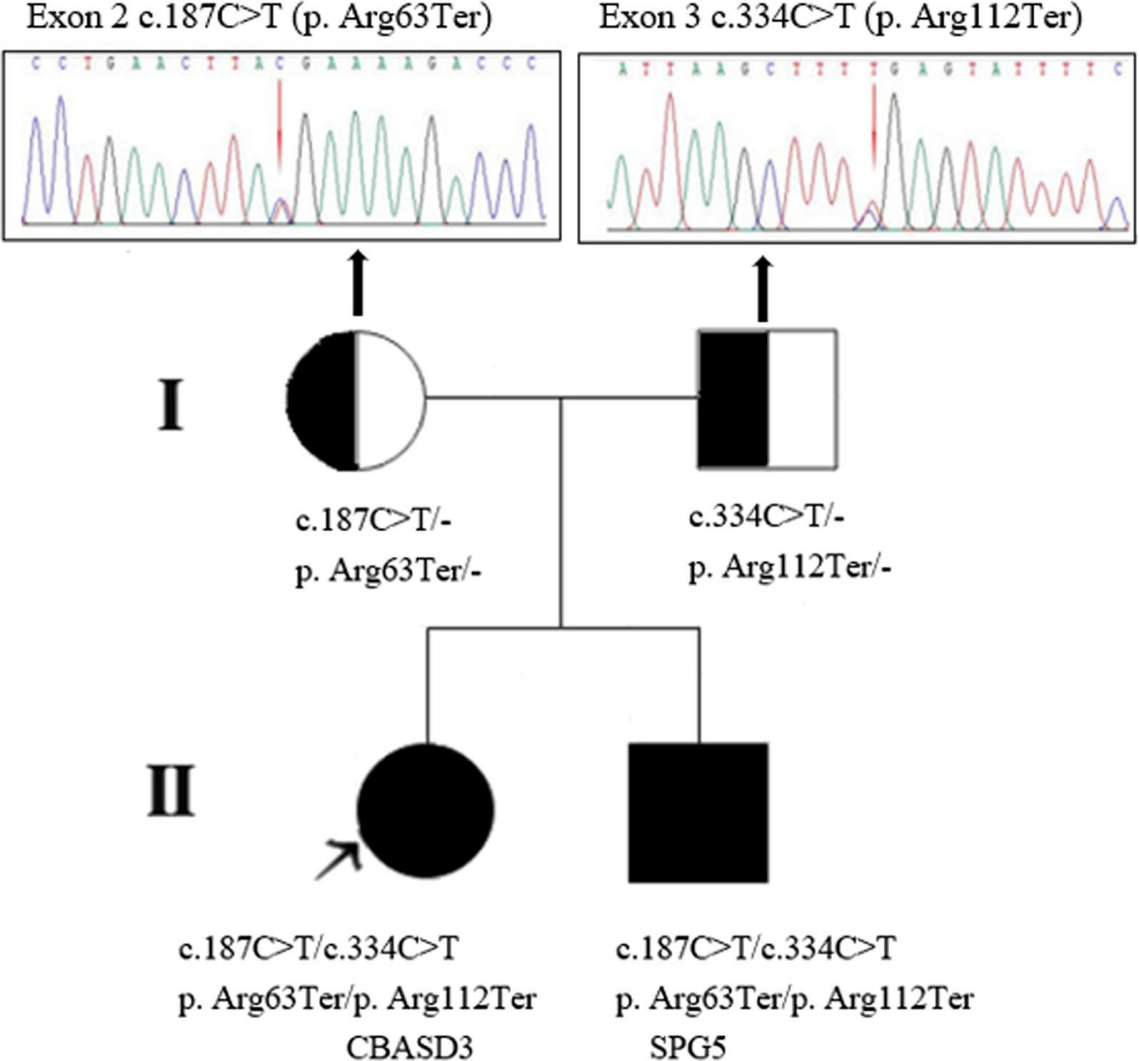
The Pedigree and two mutations in *CYP7B1*. The position of the mutant nucleotide is showed with red arrows. The congenital bile acid synthetic defect type 3 (CBASD3)- or hereditary spastic paraplegia type 5 (SPG5)-affected individuals of the family are indicated by solid symbols; the black arrow indicates index patient

The proband is the second child of a non-consanguineous couple of Chinese origin, born at 39 weeks of gestation by vaginal delivery, with a birth weight of 3.15 kg and without any perinatal problems. She developed jaundice from one month of age and received treatment with oral ursodeoxycholic acid (UDCA 5 mg/kg/d), ganciclovir for cytomegalovirus DNA 2.92 × 10^3^ copies/ml, immunoglobulin, albumin and a medium chain triglyceride-enriched formula in a local hospital, but her condition worsened and she rapidly progressed to liver failure, and had two episodes of pneumonia presenting with cough, wheezing and fever. Whole exome sequencing was performed locally, and two heterozygous mutations of c.187C > T(p.R63X)/c.334C > T(p.R112X) in *CYP7B1 *(Fig. 1) were revealed. She was admitted to Jinshan Hospital of Fudan University at the age of 5-months and 7-days old for a possible diagnosis of CBASD3.

On admission, she showed normal growth with a weight of 8.0 kg (50th–75th percentiles) and height of 65 cm (50th–75th percentiles). She was alert but not active. Physical examinations showed jaundice, grunting and tachypnoea. There were no dysmorphic features or cardiac murmur, auscultation revealed diffuse wheezing and rales in the lungs. The liver was firm and palpable at 3 cm below the right costal margin and the spleen was palpable at 4 cm below the left costal margin. Neurological examination was normal.

The laboratory investigations showed elevated levels of serum total bilirubin (TBIL), direct bilirubin (DBIL), alanine aminotransferase (ALT) and aspartate aminotransferase (AST), but normal γ-glutamyltransferase (γ-GT) (Table 1). The prothrombin time (PT) was prolonged and was only partly improved with parenteral vitamin K1 administration. Alpha-fetoprotein (AFP) was 241,863 ng/mL. The results of renal function, electrolyte levels and whole blood cell count and smear were all within normal ranges. The serological IgM of HAV, anti-HCV and HBsAg were all negative. The ultrasound of the abdomen showed hepatosplenomegaly and severe ascites.

**Table 1:** Evolution of liver and coagulation indices before and after CDCA

	Pretreatment	Starting CDCA treatment	15 days after treatment	1 months after treatment	4 months after treatment	8 months after treatment	1 year–8 months after treatment
ALT (7–40U/L)	133	127	127	104	74.9	47.4	24.6
AST (13–35U/L)	206	129	115	122	64.2	47.7	35.5
TBIL (5–21 μmol/L)	300	183	119.4	67.6	6	5.2	4.7
DBIL (0–3.4 μmol/L)	150.4	78	54.5	29.2	2.9	2.1	1.6
TBA (0–10 μmol/L)	100.9^a^	33.6^b^	54.5	65.2	12.8	4	4.9
ALP (35–135U/L)	974	742	509	536	186	236	197
γ-GT (7–45U/L)	27	26	94	183	132.7	34.1	18.4
ALB (35–45 g/L)	33	31	37	35	46.7	46.2	44.5
PT (9–12.5S)	29	19.7	17.3	15.5	11	12.8	13.3
APTT (25.4–38.4S)	75.1	47.8	46	37.9	41.7	35	34.2
INR (0.77–1.25)	2.59	1.75	1.51	1.35	0.94	0.93	1
FIB (2.38–4.98 g/L)	1.42	0.98	1.49	3.33	1.64	2.13	2.16

*ALT* alkaline aminotransferase, *AST* aspartate aminotransferase, *TBIL* total bilirubin, *DBIL* direct bilirubin, *TBA* total bile acids, *ALP* alkaline phosphatase, *γ-GT* γ-glutamyltransferase, *ALB* albumin, *PT* prothrombin time, *APTT* activated partial thromboplastin time, *INR* international normalized ratio, *FIB* fibrinogen

^a^The value during the administration of UDCA

^b^TBA level significantly decreased after UDCA stopped

She was intravenously administered albumin (7.5 g) for hypoproteinemia, and given oral supplementation of fat-soluble vitamins. Treatment of the pneumonia was with antibiotics and methylprednisolone (2 mg/kg/day). After a urine sample was collected for bile acid analysis (after 3 day’s cessation of UDCA), oral chenodeoxycholic acid (CDCA, 6 mg/kg/day) was administrated from the age of 5-months–10-days. Two weeks later, her coagulation function improved, bilirubin level decreased, and the pneumonia resolved. After discharge from hospital, methylprednisolone was gradually reduced and discontinued but she was maintained on oral CDCA. Her liver function markers, including prothrombin time nearly normalized after 8 months of CDCA therapy and she thrived well until the last follow-up at 23 months of age (Table 1). Further urine samples were collected at 4 months and 8.5 months after the onset of CDCA therapy for bile acid analysis.

## Urine bile acid analysis by FAB-MS and LC-MS/MS

The urinary bile acid profiling was analyzed at Cincinnati Children’s Hospital Medical Center. Biochemical confirmation of a bile acid synthesis disorder was obtained following screening of urine using fast atom bombardment ionization-mass spectrometry (FAB-MS) [1, 2]. Confirmation of the identity of the major urinary atypical bile acids was performed by ultra-high performance liquid chromatography electrospray ionization-tandem mass spectrometry (LC-ESI-MS/MS) using a Waters TQ-XS triple quadruple mass spectrometer interfaced with an Equity HPLC system (Milford, MA). Nitrogen was the nebulizer gas and argon the collision gas. Negative ion collision-induced dissociation (CID) spectra were obtained by direct infusion of the urine extracts and with optimized collision energy. The urine extracts were further analyzed by LC-MS/MS with chromatography on a reverse phase column and with gradient elution, essentially as described previously [13]. The reference standard of the glyco-sulfate conjugate of 3β-hydroxy-5-cholenoic acid was used to permit identification confirmation based on the CID mass spectra and HPLC retention times, and a stable-labeled analog was used for quantification of urine concentrations. Bile acid concentration was also normalized to urinary creatinine concentration.

**Fig. 2:**
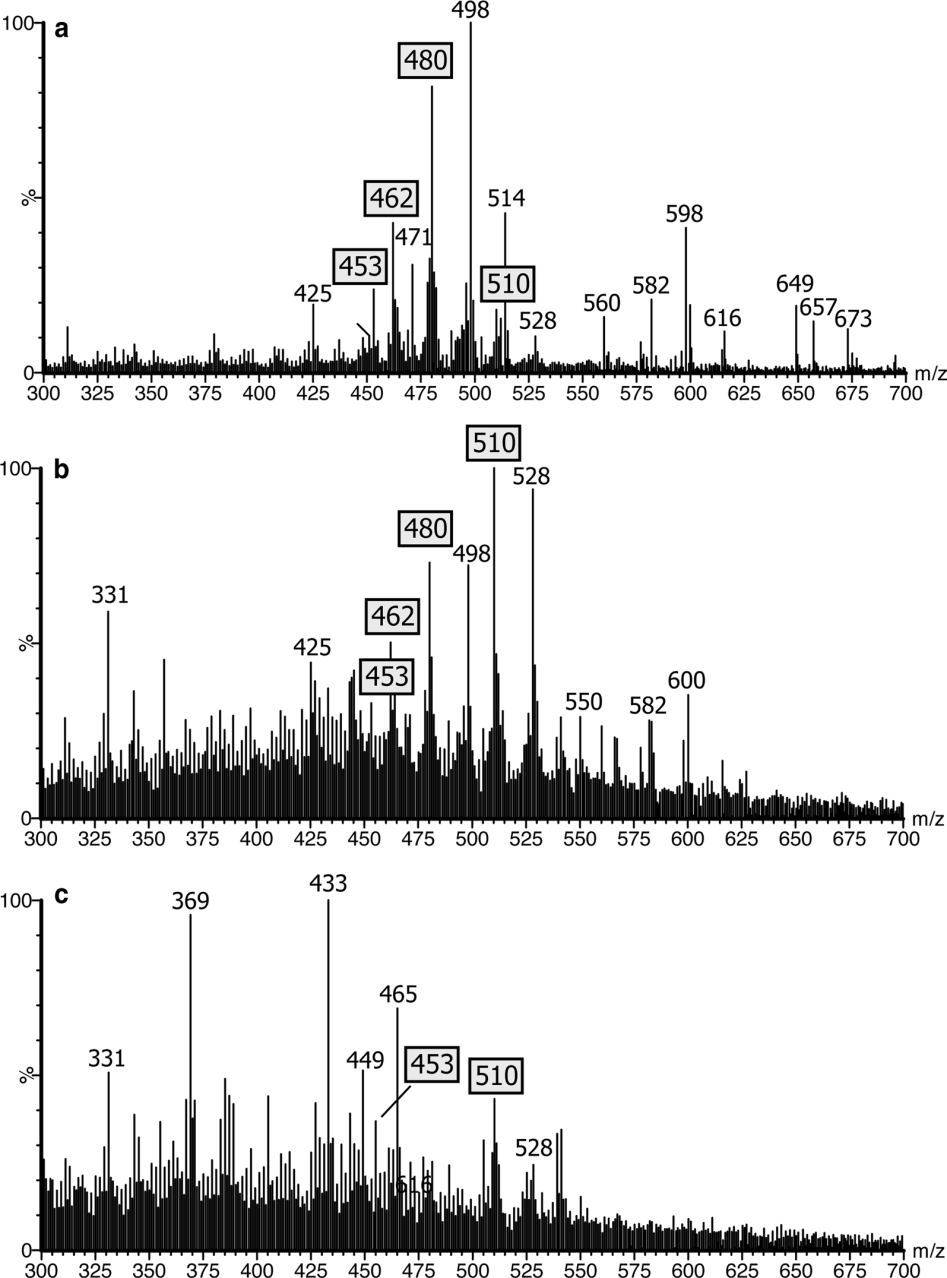
Negative ion FAB-MS spectra of the urine from the proband and her brother. **a** The mass spectrum of first urine sample collected from the proband after cessation of oral ursodeoxycholic acid; **b** the mass spectrum of the second urine sample was collected from the proband after chenodeoxycholic acid treatment for nearly 4 months; **c** the mass spectrum of the urine collected from the brother of the proband. Indicated are the specific ions that characterize the presence of elevated levels of unsaturated 3β-hydroxy-Δ^5^ bile acids associated with CYP7B1 deficiency

The profile of the first urine sample collected on admission revealed a marked elevation of bile acids consistent with significant cholestasis. The mass spectrum revealed elevated levels of glycine and sulfate conjugates of dihydroxy-, trihydroxy- and tetrahydroxy-cholanoic acids, residual to terminated UDCA therapy, and the presence of several atypical 3β-hydroxy-Δ^5^ bile acids (Fig. 2). The second urine sample collected 4 months after oral CDCA administration revealed high concentrations of atypical unsaturated monohydroxy bile acids as taurine, sulfate and glyco-sulfate conjugates. The ions at m/z of 453, 462, 480 and 510 respectively are characteristic biomarkers for a CYP7B1 deficiency [2, 3] and these represent bile acid intermediates in the alternative ‘acidic’ pathway for bile acid synthesis (Fig. 2). The classical FAB-MS profile of urinary bile acids of patients with CYP7B1 deficiency [2] in the absence of any bile acid therapy, is characterized by the absence of the normal primary bile acid conjugates and the presence of increased concentrations of hepatotoxic 3β-hydroxy-Δ^5^ bile acids [2].

**Fig. 3:**
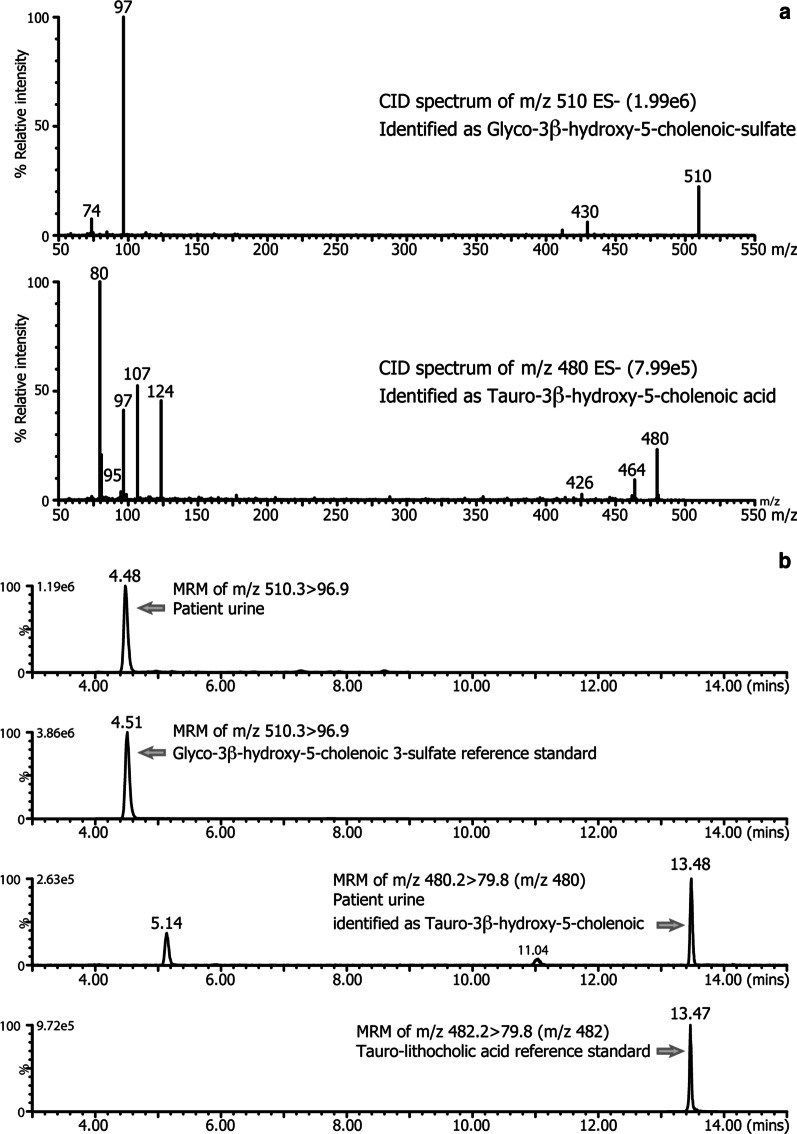
Negative ion collision-induced dissociation mass spectra of **a** the parent ions m/z 510 and m/z 480 confirming these atypical bile acids as the glyco-sulfate and taurine conjugates, respectively, of 3β-hydroxy-5-cholenoic acids. **b** The LC–MS/MS mass chromatograms of the specific ion transitions for both compounds compared with a reference standard of the glyco-sulfate conjugate of 3β-hydroxy-5-cholenoic acid and of tauro-lithocholic acid

Definitive confirmation of the structures of the atypical urinary bile acids was further established by analysis of the urine extracts using direct-flow injection and LC-MS with electrospray ionization tandem mass spectrometry (ESI-MS/MS) and collision-induced dissociation (CID) of the parent ions of m/z 510 and m/z 480, the two most abundant ions in the FAB-MS spectrum (Fig. 3). The negative ion CID mass spectrum confirmed the parent m/z 510 to be the glyco-sulfate conjugate of 3β-hydroxy-5-cholenoic acid. The daughter ion at m/z 97 arises from the cleavage and loss of sulfate, and m/z 74 from cleavage of the glycine moiety on the side-chain. This CID mass spectrum thus eliminated the possibility that m/z 510 was from the taurine conjugate of 3-oxo-7α,12α-dihydroxy-4-cholenoic acid, which has the same nominal mass, and is a biomarker for the Δ^4^-3-oxosteroid 5β-reductase (AKR1D1) deficiency [14] and often elevated in early life, or with late-stage disease. Furthermore, the HPLC retention time of m/z 510 was identical to that of a pure reference compound of this bile acid (Fig. 3). Similarly, the ion at m/z 480 was shown from its negative ion CID mass spectrum to be consistent with the taurine conjugate of 3β-hydroxy-5-cholenoic acid, and not a glycine conjugated tetrahydroxy-cholanoic acid, which would have the same nominal mass. The daughter ions at m/z 124, 107, 97 and 80 arise from cleavage of the taurine amino acid on the side-chain. The origins of these ions have been well characterized previously [15]. Although a reference compound of m/z 480 was unavailable, the LC-MS retention time and mass spectrum were found to be almost identical to that of tauro-lithocholic acid (these two bile acids would not be separated by HPLC under the conditions used) and the fragmentation pattern was similar, save an expected shift in mass of 2 Daltons for the parent ions (Fig. 3).

**Fig. 4:**
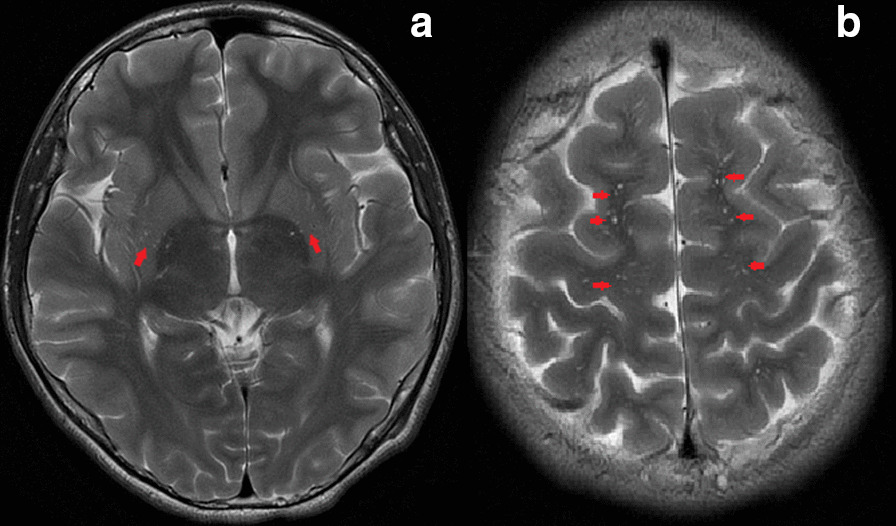
Magnetic resonance imaging (MRI) data obtained from the brother with CYP7B1 deficiency, T2-weighted images show massively enlarged perivascular spaces (showed by red arrows) in the bilateral basal ganglia (**a**) and white matter of frontal parietal (**b**)

When the proband was referred to our hospital, the family history established that she had a 15-year-old brother attending junior middle school. Apart from obvious gait abnormality and poor school sports performance evident from 13 years of age, his past history was unremarkable. He developed gait paraparesis with slowly progressive aggravation, but without intellectual or psychological problems. Neurological examination revealed hyper-reflexia and spasticity of the lower limbs, which indicated the upper motor neuron damage. His liver function markers, serum lipids, coagulation function and whole blood cell counts were all found to be normal. Brain magnetic resonance imaging (MRI) showed hyperintensity in the bilateral basal ganglia and white matter of frontal parietal on T2-weighted imaging (T2WI) which was considered as the perivascular spaces enlarged (Fig. 4). According to the clinical manifestation, physical examinations and image results, diagnosis of the pure SPG5 was suspected. The *CYP7B1* mutations identified in the proband were verified in him, and in the parents by Sanger sequencing. Compound heterozygous mutations were identified in him, and both parents were found to be heterozygous for each mutation, consistent with the autosomal recessive nature of bile acid synthesis disorders. He was then diagnosed as SPG5 and chose to be treated with CDCA (6 mg/kg/day), the same treatment used for his sister, and began rehabilitation training at the specialized physiotherapy department. After commence of the treatment, his gait abnormality and poor school sports performance did not improve. He still tripped a lot, and one time he fell on the ground and needed aid from his schoolmates to stand up. In follow up, he was reported by the parents to be non-compliant to CDCA therapy and this treatment was terminated after 2 months.

**Fig. 5:**
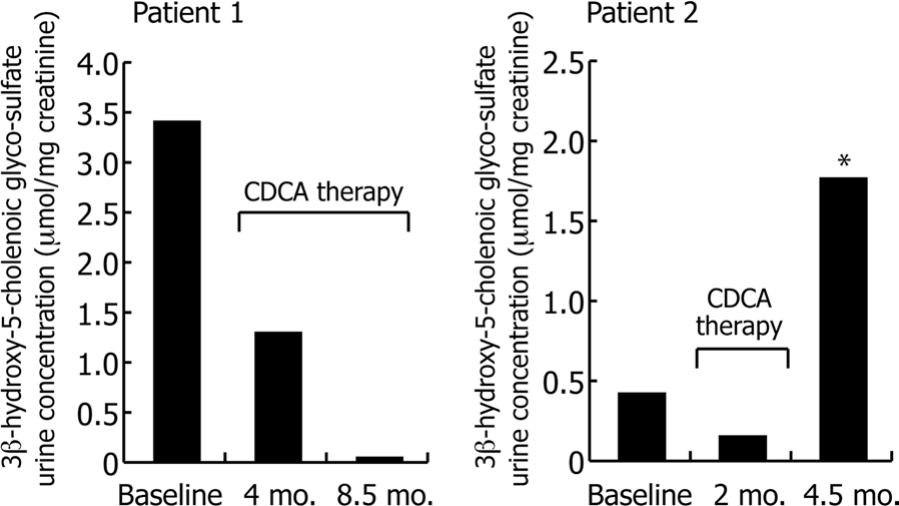
Urine bile acid concentrations normalized to creatinine of the glyco-sulfate conjugate of 3β-hydroxy-5-cholenoic acid (m/z 510 in the negative ion FAB-MS spectra) at baseline and after treatment with oral chenodeoxycholic acid in both patients with CYP7B1 deficiency. The asterix (*) denotes the patient was non-compliant and not taking chenodeoxycholic acid (CDCA) at the time of urine collection

FAB-MS analysis of his urine bile acid spectrum was unremarkable and essentially normal (Fig. 2c) reflecting absence of any cholestatic liver disease. Quantification of the urinary concentration of 3β-hydroxy-5-cholenoic acid glyco-sulfate, the major atypical urinary bile acid in CYP7B1 deficiency, confirmed marked elevations in concentration at baseline; 6.10 µmol/L for the proband and 8.8 µmol/L for the 15-year-old brother (mean ± SEM normal range for non-cholestatic patients 0.67 ± 0.18 µmol/L and for cholestatic patients 1.18 ± 0.36 µmol/L, Setchell et al. unpublished in-house data). Oral administration of CDCA (6 mg/kg/day) led to a marked decrease in the urinary excretion of 3β-hydroxy-5-cholenoic acid glyco-sulfate in both patients, consistent with suppression in hepatic synthesis. Figure 5 depicts the changes in urinary excretion of this atypical bile acid when normalized to creatinine. In the proband, the concentration of this atypical bile acid normalized by 8.5 months. However, in the 15-year-old brother, after an initial response evidenced by a decrease in atypical bile acids, the levels of 3β-hydroxy-5-cholenoic acid glyco-sulfate increased markedly when CDCA therapy ceased. These findings lend support for suppression of bile acid synthesis in the alternative (acidic) pathway by CDCA and a rebound when treatment is discontinued.

## Discussion and conclusions

Bile acids are formed from cholesterol through a series of complex enzymatic reactions in the liver. A deficiency of oxysterol 7α-hydrolase leads to the accumulation of hepatotoxic 3β-hydroxy-5-cholenoic acid and reduced levels of primary bile acids, the essential driving force for bile flow, which collectively leads to progressive cholestasis. Oxysterol 7α-hydroxylase deficiency was first described in 1998 by Setchell et al. [2], presenting as neonatal cholestasis (CBASD3). Only 6 more cases have been reported thereafter (Table 2) [3, 4, 16–18]. With so few described patients, knowledge of the natural history of CSASD3 is limited. Consistent with the genetic analysis, the detection of increased concentrations of 3β-hydroxy-Δ^5^ bile acids in the urine confirmed CYP7B1 deficiency in the proband as another new case of CBASD3.

**Table 2:** Clinical and laboratory features of the 8 patients with neonatal cholestasis caused by oxysterol 7α-hydroxylase (CYP7B1) deficiency

Patient	Race	Sex	Age at first diagnosis	Urine mass spectrometry—abnormal features	Organomegaly	*CYP7B1* mutation	Treatment and outcome
Setchell et al. [2]	Hispanic	M	10 weeks	Elevated 3β-hydroxy-5-cholenoic acid	Hepatomegaly	R388X/R388X	CA 15 mg/kg/day, but no response after 49 d, CDLT at 4.5 mo, died at 5 mo
Ueki et al. [16]	Chinese	M	5 months	Elevated 3β-hydroxy-5-cholen-24-oic acid	Hepatosplenomegaly	R112X/R112X	UDCA, died at 11 mo
Mizuochi et al. [17]	Japanese	F	6 months	Elevated 3β-hydroxy-5-cholen-24-oic acid	Hepatosplenomegaly	R112X/R417C	LDLT at 8 mo, alive (29 mo)
Dai et al. [3]	Pakistani	M	3–4 months	Elevated 3β-hydroxy-5-cholenoic acid	normal	R417C/R417C	CDCA 15 mg/kg/day firstly, improved after 2 d, normal liver function by 7 mo, CDCA 6 mg/kg/day at 5 years, alive (6.5 years)
Jeana et al. [18]	Korean	M	3 months	Elevated 3β-hydroxy-Δ^5^-bile acids	Hepatosplenomegaly	R338X/Y469Ifs	LDLT at 4 mo, alive (33 mo)
Ju-Yin Chen et al. [4]	Chinese	M	4 months	Elevated 3β-monohydroxy-Δ^5^-bile acids	NR	R112X/R112X	CDCA 9 mg/kg/day, liver function deteriorated, LT at 1 year, died at 1 year
Ju-Yin Chen et al. [4]	Chinese	M	3 months	Elevated 3β-monohydroxy-Δ^5^-bile acids	Hepatosplenomegaly	R112X/R112X	CDCA 15 mg/kg/day firstly, liver function improved after 2 mo, CDCA 5 mg/kg/day at mo, alive (3 years)
Present case	Chinese	F	5 months	Elevated 3β-hydroxy-Δ^5^-bile acids	Hepatosplenomegaly	R63X /R112X	CDCA 6 mg/kg/day, liver function improved after 7 d, alive (23 mo)

*M* male, *F* female, *NR* not reported, *ND* no data, *UDCA* ursodeoxycholic acid, *CA* cholic acid, *CDCA* chenodeoxycholic acid, *CDLT* cadaveric donor liver transplantation, *LDLT* living donor liver transplantation

Table 2 summarizes the main features of all these published cases of CBASD3, together with the patient described here. In all, there were 5 males and 3 females, all presenting with prolonged jaundice and/or hepatosplenomegaly from between 6 days and 5 months after birth. All the infants were characterized by elevated conjugated bilirubin and transaminases, but normal γ-GT. All the patients had poor responses to UDCA therapy, including one patient that died of liver failure at the age of 11 months [16], and 2 patients that were liver transplanted at the age of 4 months [18], and 8 months [17] respectively. The others were treated with other medications after a diagnosis of CBASD3 was made. Based on these reports, UDCA clearly has no effect on the clinical course, partly because it does not inhibit bile acid synthesis and the production of atypical bile acids continues unabated.

Apart from our case, two other CBASD3 patients completely recovered after CDCA replacement therapy. One patient started CDCA (15 mg/kg/day) at the age of 4.5 months and liver function tests returned to normal by 7 months of age [3]; the other patient started CDCA (15 mg/kg/day) at 3 months of age and the cholestasis resolved by age 5 months [4]. One treatment failure was reported in a patient that started CDCA (9 mg/kg/day) from 4 months of age, and then underwent liver transplantation at 1 year of age [4].

To date, only one patient has been treated with cholic acid (CA 15 mg/kg/day), for 49 days from 11 weeks of age, but then underwent cadaveric liver transplantation at the age of 4.5 months because of disease progression [2]. It is difficult to say with certainty that this primary bile acid is not helpful for patients with CBASD3.

Liver transplantation is believed the only choice for patients with CBASD3 who do not recover after drug treatment [16]. But the complications of the operation, infection, graft versus host disease, side-effects of long-term use of immunosuppressant impact prognosis. A total of four patients with CBASD3 have undergone liver transplantation [2, 4, 17, 18] and two died from complications; one from Epstein-Barr virus-related disseminated lymphproliferative disease on postoperative day 20 [2], the other from an intestinal perforation and sepsis on postoperative day 17 [4].

Why cholic acid failed, albeit in only one case, and chenodeoxycholic acid has proven helpful is difficult to explain. It may be that CDCA suppresses sterol 27-hydroxylase, the first step in the acidic pathway for bile acid synthesis, leading to decreased formation of atypical 3β-hydroxy-5-cholenoic acids, alleviating the liver damage and promoting bile flow. This would be supported by our evidence for reductions in the urinary concentrations of the major atypical bile acid in these patients, and the rebound in levels when there occurred non-compliance to therapy in the older child. The patient we described is the third case to date that has responded to treatment with CDCA.

Our observations also show that the phenotype of *CYP7B1* deficiency is highly variable, even within siblings. In this report the sister had cholestasis early in life, while her brother with the same mutations in *CYP7B1* developed the typical clinical features of SPG5, but without the liver disease. It therefore appears that there is no obvious correlation between the phenotype and genotype in *CYP7B1* deficiency. Stiles et al. proposed the different manifestations of *CYP7B1* defects may be caused by the accumulation of different *CYP7B1* substrates in the body, leading to different clinical features [19]. However, a couple of siblings, who were diagnosed as SPG5 with compound heterozygous mutations (c.333_334delTC and c.806delA) as adults, were described as having a past history of prolonged neonatal jaundice [20]. So it is possible that a CBASD3 infant that survives after CDCA therapy may suffer from SPG5 later.

There is no effective therapeutic strategy to improve the neurological disorder of patients with SPG5. In one report, CDCA treatment for SPG5 was reported to increase serum CDCA, lithocholic and UDCA, and decrease cholic acid and deoxycholic acid, but long-term studies are needed to draw conclusions as to its effectiveness [20]. The 15-year-old brother with SPG5 in this report was treated with CDCA for only 2 months. Although his second urinary bile acid profile was near normal, his neurological symptoms progressed, suggesting CDCA, at least in the short term, was not helpful.

Based on our observations of these two cases, we conclude that the bile acid profile of urine, and the clinical manifestations in cases of CYP7B1 deficiency vary widely, even within the same family. We believe that CDCA maybe the appropriate choice of therapy of this deficiency when presenting as liver disease, and this may circumvent the need for liver transplantation.

## Data Availability

The datasets used and/or analyzed during the current study are available from the corresponding author on reasonable request.

## Abbreviations

CBASD: Congenital bile acid synthesis deficiencies
CBASD3: Congenital bile acid synthesis defect type 3
CYP7B1: Oxysterol-7α-hydroxylase
SPG5: Hereditary spastic paraplegia type 5
UDCA: Ursodeoxycholic acid
TBIL: Total bilirubin
DBIL: Direct bilirubin
ALT: Alkaline aminotransferase
AST: Aspartate aminotransferase
γ-GT: γ-Glutamyltransferase
PT: Prothrombin time
AFP: Alpha-fetoprotein
CDCA: Chenodeoxycholic acid
FAB-MS: Fast atom bombardment mass spectrometry
LC-ESI-MS/MS: Liquid chromatography electrospray ionization-tandem mass spectrometry
CID: Collision-induced dissociation
MRI: Magnetic resonance imaging
T2WI: T2-weighted imaging
TBA: Total bile acids
ALP: Alkaline phosphatase
ALB: Albumin
APTT: Activated partial thromboplastin time
INR: International normalized ratio
FIB: Fibrinogen
CA: Cholic acid
